# Maxillary Teeth Abscesses Result in Atypical Liver Abscesses

**DOI:** 10.7759/cureus.2353

**Published:** 2018-03-20

**Authors:** Ellecia Rainwater, Vritti Gupta, Renuga Vivekanandan, Gary Gorby

**Affiliations:** 1 Department of Internal Medicine, CHI Health, Creighton University; 2 Department of Infectious Disease, CHI Health, Creighton University; 3 Creighton University, Veterans Affairs Omaha

**Keywords:** dentistry and oral medicine, healthcare improvement and patient safety, medical education, liver abscesses

## Abstract

Hepatic liver abscesses are often misdiagnosed on initial presentation because pyogenic liver lesions are a rare occurrence in the United States. This leads to a delay in proper treatment and results in increasing morbidity and mortality. Our case report demonstrates the atypical presentation of a hepatic liver abscess in the elderly. The source of infection was found to be periapical abscesses of the teeth, which subsequently seeded the portal blood stream of our patient. Our findings validate the potential hazard of Viridans streptococci and illustrate how untreated dental infections can serve as a reservoir for a systemic infection.

## Introduction

In the United States, the incidence of pyogenic liver abscess has increased from 2.7 to 4.1 per 100,000 population from 1994 to 2005 [1]. This rise in incidence could be attributed to an aging population, an increase in instrumental modalities to view the hepatobiliary tree, and an increase in hepatobiliary disease, metabolic syndromes, and liver transplantations [1]. The most common isolated pathogens, without a history of travel abroad or recent immigration to the United States, are Streptococcus species and Escherichia coli [1].

There are a limited number of case reports identifying the members of the Streptococcus milleri group as the etiology of multiple liver abscesses. The organisms Streptococcus intermedius and Streptococcus constellatus, members of the Streptococcus milleri group, typically colonize in the oropharynx, genitourinary tract, and gastrointestinal tract [2]. These bacteria are normally commensal but can cause pathological abscesses in the myocardium, pleural cavity, or in organs where surgical or endoscopic manipulation has occurred [3]. Such infections are a direct result of hematogenous spread or a direct extension of an intra-abdominal liver infection through damaged mucosa, which serves as a gateway for the bacteria to disseminate and seed the portal blood stream [2,3].

## Case presentation

**Figure 1:**
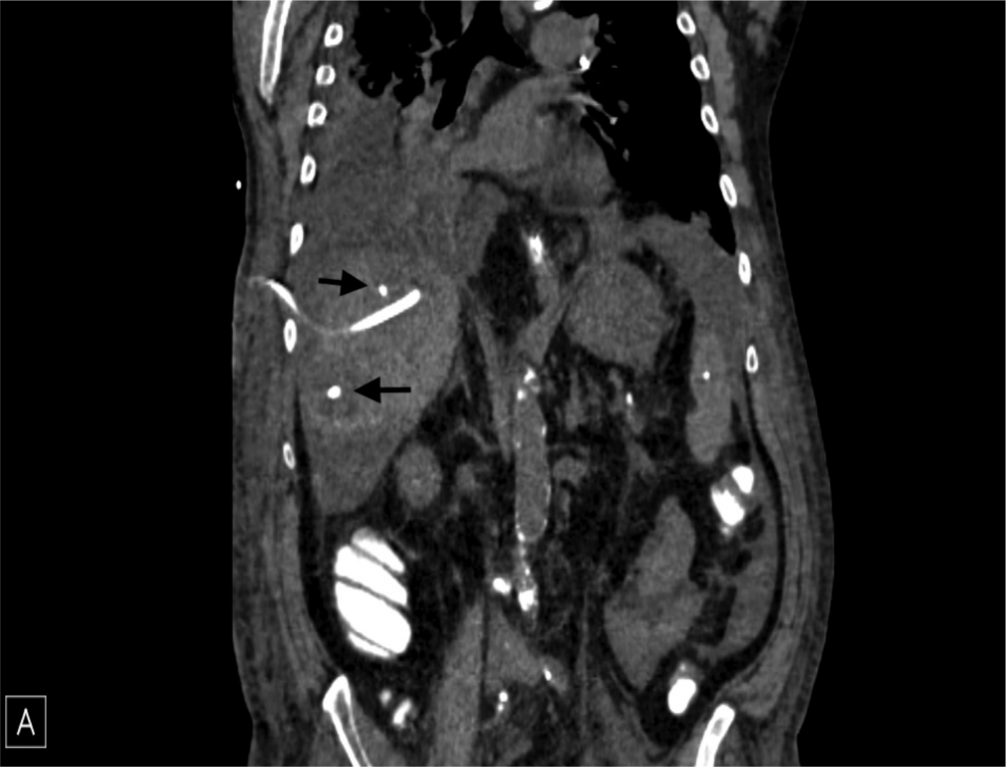
Computed tomography scan of the abdomen/pelvis without contrast showing intrahepatic drains near the hepatic abscesses.

A 69-year-old male veteran with a past medical history of a right orchiectomy for localized testicular seminoma presented with left lower abdominal pain initially thought to be due to constipation. Bowel regimens failed to relieve symptoms and prompted a return visit. His symptoms had progressed to non-bilious emesis, several episodes of non-bloody diarrheal stools, fevers, oliguria, increasing dyspnea, and poor oral intake. His social and occupational history was significant for military service in Vietnam with exposure to Agent Orange and a 50-pack year history of smoking. His physical exam was significant for rebound tenderness, right upper quadrant (RUQ) tenderness, and lack of jaundice. His laboratory results were significant for a white blood cell count of 23.4 K/uL (4.5 K/uL – 10 K/uL), procalcitonin 19.51 ng/mL (3 ng/mL – 15 ng/mL), alanine aminotransferase 86 IU/L (7 IU/L – 56 IU/L), aspartate aminotransferase 117 IU/L (10 IU/L – 40 IU/L), total bilirubin 1.4 mg/dL (0.1 mg/dL – 1.3 mg/dL), albumin 2.9 g/dL (3.5 g/dL – 5.5 g/dL), and international normalized ratio of 1.3 (0.8 – 1.1) in the absence of warfarin. Serum beta human chorionic gonadotropin and alpha-fetoprotein were within normal laboratory range suggesting that the testicular seminoma was successfully treated. Two sets of blood cultures did not grow any bacteria or fungi. An abdominal ultrasound revealed a 6 cm liver mass, and a computed tomography (CT) scan confirmed two hepatic lesions: 6.3 cm x 5.6 cm and 4.9 cm x 3.9 cm, respectively. Figure 1 shows the hepatic drains interventional radiology (IR) placed to help drain the abscesses.

**Figure 2:**
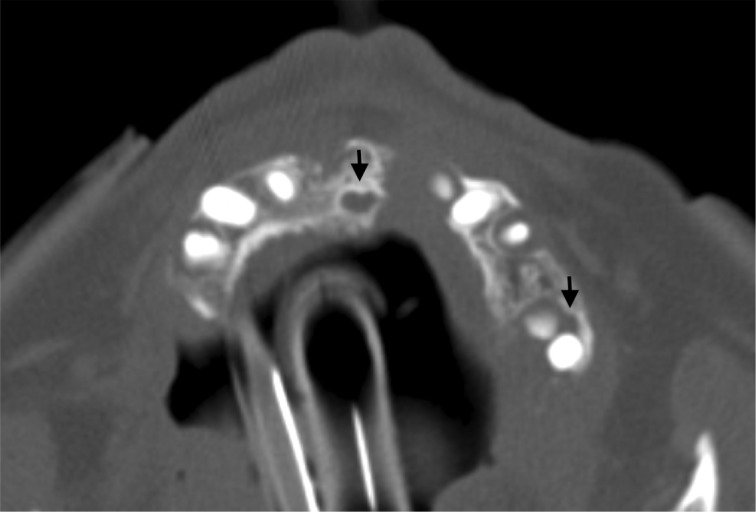
Computed tomography maxillofacial imaging demonstrating large periapical abscesses adjacent to the maxillary teeth.

IR-guided drainage of the abscesses was complicated by abscess rupture, hypotension, and septic shock resulting in endotracheal intubation and initiation of vancomycin and meropenem. Cultures from the hepatic abscesses revealed a polymicrobial infection with Streptococcus intermedius and Streptococcus constellatus. Subsequently, vancomycin was discontinued and meropenem was changed to ertapenem as coverage for pseudomonas was no longer warranted. As the isolated pathogens typically colonize the oropharyngeal region, further investigations focused on the thoracic and facial regions. A transesophageal echocardiogram was unremarkable for vegetations or shunts; however, a maxillofacial CT scan (Figure 2) revealed multiple maxillary teeth abscesses.

Unfortunately, the patient passed away due to worsening septic shock. Furthermore, because the patient’s family declined autopsy, no tissue was available to confirm the source of infection.

## Discussion

Due to their relatively low prevalence in the elderly population, hepatic abscesses are frequently omitted as a cause of abdominal pain by health care professionals. However, this trend appears to be shifting as the annual incidence of hospitalization for pyogenic liver abscess in the United States is rising [1,4]. Patients over the age of 65 are 10 times more likely to acquire a pyogenic liver abscess than younger patients, and men are at an increased risk for developing a pyogenic liver abscess as compared to women across all age groups [4]. A missed hepatic abscess results in a delayed treatment likely leading to increased morbidity and mortality in this fragile population.

Clinical presentation of hepatic lesions varies based on the location and size of the abscess. The classic triad of fever, RUQ pain or fullness, and jaundice is rarely seen. Instead, a broad range of symptoms including nausea, vomiting, malaise, and/or weight loss may be the presenting chief complaint(s) [5]. Infections from the Streptococcus species tend to follow this indolent course of nonspecific symptoms, which may result in a failure to identify the source of infection.

Due to vague presenting symptoms, laboratory values may be required to suggest a diagnosis [5]. Typically, hypoalbuminemia, elevated transaminases, and leukocytosis are prominent in patients with hepatic lesions [5]. The initial diagnostic test used to assess RUQ abdominal complaints is an ultrasound. However, CT imaging has increased sensitivity and thus is the most accurate way to diagnose a pyogenic liver abscess [4,5]. Although imaging modalities are sensitive to confirm the pathology of a liver abscess, microbiological analysis is essential to identify the causative agent(s) and to inform the proper antibiotic management plan [5]. Prior to cultures, initial antibiotic treatment is centered on a broad-spectrum polymicrobial approach and the regimen tailored as the culture reports are finalized. Although multiple antibiotic regimens exist, they should include an extended spectrum βeta-lactam or a combination of a third-generation cephalosporin or fluoroquinolone and metronidazole [6]. After extensive investigation, the periapical tooth abscesses were considered to be the leading source of the liver abscesses in the absence of an autopsy. The pathogens Streptococcus intermedius and Streptococcus constellatus cultured from the patient’s liver abscesses are commensal mouth flora. The infectious and inflammatory processes surrounding the maxillary teeth may have provided a portal of entry into the venous system and then subsequently seeded the hepatic artery by the arterial circulation.

This case illustrates the potential ramifications of untreated dental infections and explores the need for preventing dental infections in order to prevent systemtic infection and/or liver abscesses. Neumayr el al. demonstrated a clinical improvement (defined as decreasing leukocytosis and C-reactive protein) in an 18-year-old immunocompetent patient with multiple liver abscesses after the source, an infected molar, was extracted [7]. Furthermore, sonographic imaging of the liver abscesses showed regression of the abscesses’ cavity sizes after extraction of the source [7]. Unfortunately, source control was not obtained in our patient as he passed away before dental extraction and culture of the periapical abscesses were performed.

Additional review of the literature validates the importance of proper oral hygiene care and the potential trauma dental manipulation can cause. In a study by Whiley et al., Streptococcus intermedius was identified in most of the isolated strains from dental plaques and associated with liver or brain abscesses [8]. Livingston and Perez-Colon presented the first case of a pyogenic liver abscess related to Streptococcus intermedius in a 65-year-old male after a routine dental cleaning [2]. Tran and colleagues reported two cases of invasive Streptococcus intermedius infection causing multiple hepatic abscesses in two previous healthy individuals [9]. These cases predominantly report a monomicrobial infection instead of a polymicrobial infection resulting in liver abscesses. In our case, however, two species of the Streptococcus milleri group—Streptococcus intermedius and Streptococcus constellatus—were isolated from the patient’s lesions. In addition, most of the previous reports did not result in patient mortality as the patients were considerably younger than our veteran and suggests the fragility of the elderly population [2].

It is important to realize that a pyogenic liver abscess is confirmed or suspected at the time of admission in less than one third of the cases [4]. Often an intra-abdominal process is not even considered in the differential due to the nonspecific clinical presentation until after the patient fails to respond to medical therapy and additional ultrasound or CT is ordered [4]. This delay in appropriate treatment of pyogenic liver abscesses results in significant morbidity and mortality as demonstrated by our case report.

## Conclusions

This case serves to highlight the importance of having a low threshold of suspicion in an elderly patient presenting with atypical abdominal pain and concerning laboratory values (e.g., hypoalbuminemia, elevated transaminases, and/or leukocytosis). It is important to perform a thorough physical exam (e.g. oral mucosa exam) to evaluate for all possible etiologies for infectious sources. Poor dental hygiene although seemingly benign can lead to significant ramifications in the elderly. Physicians should also consider imaging to rule out liver lesion(s) in these patients to prevent complications and fatal decompensation in the elderly population.
